# A deep learning-based cascade algorithm for pancreatic tumor segmentation

**DOI:** 10.3389/fonc.2024.1328146

**Published:** 2024-08-07

**Authors:** Dandan Qiu, Jianguo Ju, Shumin Ren, Tongtong Zhang, Huijuan Tu, Xin Tan, Fei Xie

**Affiliations:** ^1^ School of Information Science and Technology, Northwest University, Xi’an, Shaanxi, China; ^2^ Department of Radiology, Kunshan Hospital of Chinese Medicine, Kunshan, Jiangsu, China; ^3^ College of Computer Science and Technology, Xidian University, Xi’an, Shaanxi, China

**Keywords:** pancreatic tumor segmentation, cascaded algorithm, deep learning, non-local localization module, focusing module

## Abstract

Pancreatic tumors are small in size, diverse in shape, and have low contrast and high texture similarity with surrounding tissue. As a result, the segmentation model is easily confused by complex and changeable background information, leading to inaccurate positioning of small targets and false positives and false negatives. Therefore, we design a cascaded pancreatic tumor segmentation algorithm. In the first stage, we use a general multi-scale U-Net to segment the pancreas, and we exploit a multi-scale segmentation network based on non-local localization and focusing modules to segment pancreatic tumors in the second stage. The non-local localization module learns channel and spatial position information, searches for the approximate area where the pancreatic tumor is located from a global perspective, and obtains the initial segmentation results. The focusing module conducts context exploration based on foreground features (or background features), detects and removes false positive (or false negative) interference, and obtains more accurate segmentation results based on the initial segmentation. In addition, we design a new loss function to alleviate the insensitivity to small targets. Experimental results show that the proposed algorithm can more accurately locate pancreatic tumors of different sizes, and the Dice coefficient outperforms the existing state-of-the-art segmentation model. The code will be available at https://github.com/HeyJGJu/Pancreatic-Tumor-SEG.

## 1 Introduction

Pancreatic cancer is a malignant tumor of the digestive tract characterized by poor therapeutic effects and unfavorable prognosis. Although this cancer currently accounts for a mere 3% of total cancers in the United States, its malignancy remains the fourth leading cause of cancer deaths in the United States (1). Pancreatic cancer patients have a 26% five-year survival rate if the cancer is not metastatic and localized after diagnosis, and only 2% otherwise (2). Unfortunately, the pancreas is hidden behind the liver and intestines, and conventional ultrasound examinations are limited to the detection of pancreatic tumors larger than 3 cm. This constraint poses challenges for achieving early diagnosis. If the tumor is malignancy, it frequently manifests at an advanced stage, accompanied by metastasis to other anatomical regions of the body (3). Therefore, early localization and measurement of the location and extent of pancreatic tumors (*i.e.*, segmentation) are crucial for the diagnosis and radiotherapy of advanced pancreatic cancer. In clinical practice, radiologists manually delineate the boundaries of pancreatic tumors in medical images following clinical experience. Given that one single abdominal scan of a patient typically comprises 100 to 200 slices at 5mm intervals, annotating these slices would directly escalate the workload and time cost for radiologists. In addition, the subjective factors among different radiologists may result in disparities in the labeling results for identical medical images, leading to deviations in the treatment plan. Inappropriate treatment plans can lead to delays in the condition and cause patients to miss the optimal time for timely intervention. To reduce the burden on doctors and improve the accuracy and objectivity of pancreatic tumor recognition, certain scholars have conducted research on automatic tumor lesion segmentation based on Deep learning.

**Figure 1:**
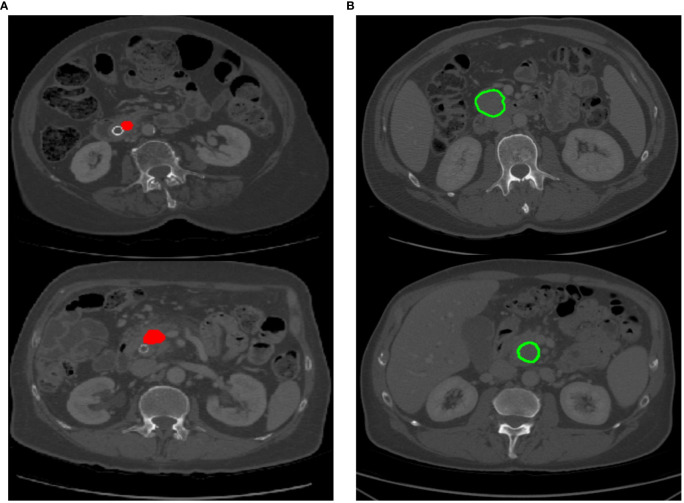
Different labeling patterns for pancreatic tumors. **(A)** Tumor area labeling; **(B)** Tumor contour labeling.

Several efforts have been attempted for automating organ or lesion segmentation, especially regularly-shaped and large-area targets (4–7) such as lungs, liver, and heart. These efforts have achieved exciting performance. There are limited studies on pancreatic tumor segmentation, and existing works (8–11) mainly rely on attention modules or prior cues to enhance the feature representation of the entire 2D or 3D pancreatic tumor images. Nonetheless, these methods primarily emphasize the salient regions within the image, making it challenging to pay attention to the local details of the target. This is mainly because (1) As shown in **Figure 1A**, the red highlighted area represents the pancreatic tumor area, which accounts for a small proportion of the input image. This leads to the network being easily confused by complex and variable background information during training, resulting in inaccurate tumor localization. (2) As shown in **Figure 1B**, the green contour line represents the boundary of the pancreatic tumor. The pancreatic tumor exhibits low contrast with the surrounding background, and the boundary line is indistinct, resulting in challenges such as false positives and undersegmentation issues during the pancreatic tumor segmentation process. To accurately locate and measure pancreatic tumors from medical images, clinical doctors usually first determine the location of the pancreas to ascertain the approximate range of a pancreatic tumor. Subsequently, they carefully identify and reduce the range until a clear pancreatic tumor is identified. Inspired by the doctor’s diagnostic process, we propose a cascaded automatic neural algorithm for pancreatic tumor segmentation, which consists of two stages to gradually segment tumor targets. The prediction probability map in the first stage reduces the input scale in the second stage, effectively reducing substantial background interference. These two stages are connected by salient change modules and are jointly optimized through gradient backpropagation to sequentially segment the pancreas and pancreas tumors. In the second stage, we further carefully design a multi-scale pancreatic tumor segmentation network based on non-local localization and focusing modules on solving the under-segmentation or false-positive pancreatic tumors. Specifically, we use a multi-scale U-Net network as the backbone to extract and fuse multi-level features of pancreatic tumors, and further input these features into five convolutional layers to reduce channels. Then, we use our non-local localization module at the highest level of the encoder to locate the approximate area of pancreatic tumors. Finally, we design multiple focusing modules to gradually detect and eliminate false negative and false positive interference. We find that the low proportion of small-scale pancreatic tumors in the Loss function is the main reason for the missed segmentation of pancreatic tumors. Therefore, we introduced a Loss function based on the shared boundary between classes to improve the contribution of small-scale pancreatic tumors to network training. Our main contributions are summarized as follows:

- We propose a novel cascaded automatic neural algorithm for the pancreatic tumor segmentation task. This algorithm contains two stages, which are jointly optimized by gradient backpropagation during training.
- We employ multi-scale U-Net as the backbone to obtain deeper feature representations of pancreatic tumors. In the second stage, we carefully designed a non-local localization module and a focus module to capture target detail feature representations, alleviating the under-segmentation and false positives problems.
- We use the Sørensen–Dice coefficient, sensitivity, and specificity to perform comparative experiments with other state-of-the-art methods on the pancreatic tumor segmentation dataset and pancreas segmentation dataset. The experimental results show that our algorithm exhibits the most superior performance in both segmentation evaluation indicators and visual effects.

## 2 Related work

In the past decade, Deep learning algorithms have made great achievements in the field of computer vision, especially in computer-aided diagnosis (CAD). Segmentation of various organs and tumors using medical images is always a mainstream task in CAD. Due to the intuitive representation of tissues and organs through medical images, high-quality medical images play an important role in disease diagnosis. At present, there are various medical imaging methods. Computed Tomography (CT) (12) and Nuclear Magnetic Resonance Imaging (NMRI) (13) can clearly reflect the anatomical structure of various tissues in the human body, and are usually used in tumor diagnosis. Due to the sensitivity of CT images to the area where the lesion occurs, they can display the location, size, and whether the tumor has metastasized. So the application of CT images is more extensive.

At present, there has been a lot of research on medical image segmentation tasks. The U-Net (14) adopted an encoder-decoder structure and high-low feature skip connections, which can achieve excellent segmentation results from a small amount of training data. So the model was very suitable for medical image segmentation. However, the fatal weakness of this Convolutional neural network is that it pays equal attention to the features extracted from the image. This problem results in the same impact of input features on the model, causing the model to be unable to focus directly on the target and reducing the rate of convergence of the model. Some works introduced attention mechanisms to improve the accuracy of medical image segmentation models. Ashish Sinha et al. (15) utilized a self-attention mechanism to capture rich contextual information and construct a dependency relationship between local and global features. The model achieved better segmentation performance in abdominal organs, cardiovascular structures, and brain tumor segmentation experiments. Guimin Hou et al. (16) proposed a medical image segmentation network based on attention mechanism and feature fusion. A dual attention module composed of parallel channels and spatial attention branches was added to the backbone network to adaptively calibrate and weight features, achieving the highest segmentation accuracy in the segmentation experiment of the aorta and lung. Bangcheng Zhan et al. (17) designed a multi-attention News aggregator to aggregate globally effective features by fusing multi-level local attention. This model solved the problem that the net paid too much attention to the features of interest while ignoring the secondary salient features, and obtained excellent evaluation results on the medical image segmentation dataset. There were many other medical image segmentation tasks that incorporated attention methods, such as (18–20). Although these models based attention have achieved good results, they were all designed for segmentation of large organs such as the lungs (21), heart (22), liver (23), brain (24) or well-defined tumors. Medical image segmentation tasks with small target scales, blurred boundaries, and high background similarity such as pancreatic tumors do not perform well.

Recently, some researchers have designed some models for the segmentation of pancreatic tumors. Taleb et al. (9) proposed five different 3D self-supervised methods for pancreatic tumor segmentation in order to learn more features from unlabeled 3D images and reduce the cost of manual annotation. Wang et al. (25) proposed a model that took a 2D network as the backbone, used an attention mechanism as the bridge to train classifiers, and used Inductive Attention Guidance Network (IAG-Net) to obtain the segmentation results of pancreatic cancer. It achieved image-level and pixel-level segmentation. Zhu et al. (26) used a pancreatic tumor segmentation network with multiple different input scales, with sizes of 64^3^ 32^3^ and 16^3^, respectively. The network adopted a coarse and fine segmentation strategy. This strategy first used a network with an input image size of 64^3^ for coarse segmentation and then used a network with input scales of 32^3^ and 16^3^ to search for small pancreatic tumors that may have been missed in the coarse segmentation results. Simultaneously the network introduced non parametric post-processing algorithms to remove erroneous segmentation results. These three methods were all one-stage networks, although they showed some improvement in the segmentation effect of pancreatic tumors. However, pancreatic tumors of the small scale only occupy a small part of CT images and have high variability in shape and position. These one-stage networks cannot accurately locate pancreatic tumors, thus affecting the segmentation results. Therefore, some researchers proposed the two-stage segmentation algorithm. Jie Xue et al. (27) proposed a cascaded multitasking 3D fully convolutional network (FCN) for automatically segmenting the pancreas. The network consisted of two parts. The first part focused on quickly locating the pancreatic region, while the second part used a multi-task FCN with dense connections to refine the segmented image for fine pixel segmentation. Qi Li et al. (28) proposed a 3D full Convolutional neural network with three temperature guidance modules, namely, balance temperature loss, rigid temperature optimizer, and soft temperature indicator, to achieve pancreatic tumor segmentation. The rigid temperature optimizer utilized the Metropolis principle to guide the global training of the network. When the model was stable, the training loss and learning rate were updated to optimize the network according to the soft temperature indicator. 3D networks can combine information between image layers to ensure the continuity of changes between image masks, thus improving performance to a certain extent. However, the 3D network limits the maximum receptive field of the network, losing some global information. This type of network occupies too much video memory, resulting in the conventional GPU limiting the training and testing of the network, and hindering the further application of clinical diagnosis. There was little research on the two-stage pancreatic tumor segmentation algorithm based on 2D. Zhou et al. (29) proposed a two-stage network for pancreatic tumor segmentation based on the high correlation between pancreatic and pancreatic tumor locations, which segmented pancreatic tumors on the basis of relatively easy pancreatic segmentation tasks. The network consisted of two stages. The first stage segmented the pancreas. The second stage segmented pancreatic tumors and the first stage provided location information for the second stage. However, when optimizing the network, the segmentation models of the two stages were optimized separately, resulting in inconsistent training and testing processes. To solve this problem, we connect the two stages through a saliency change module and use gradient backpropagation for joint optimization to improve their respective segmentation performance. In addition, on the basis of adding channel attention and spatial attention, we improve the multi-scale feature extraction network to extract multi-scale pancreatic tumor features. In order to alleviate the problem that small targets have relatively little impact on model training, we improve the overall Loss function and increase the contribution of small pancreatic tumors to the Loss function.

## 3 Methods

**Figure 2:**
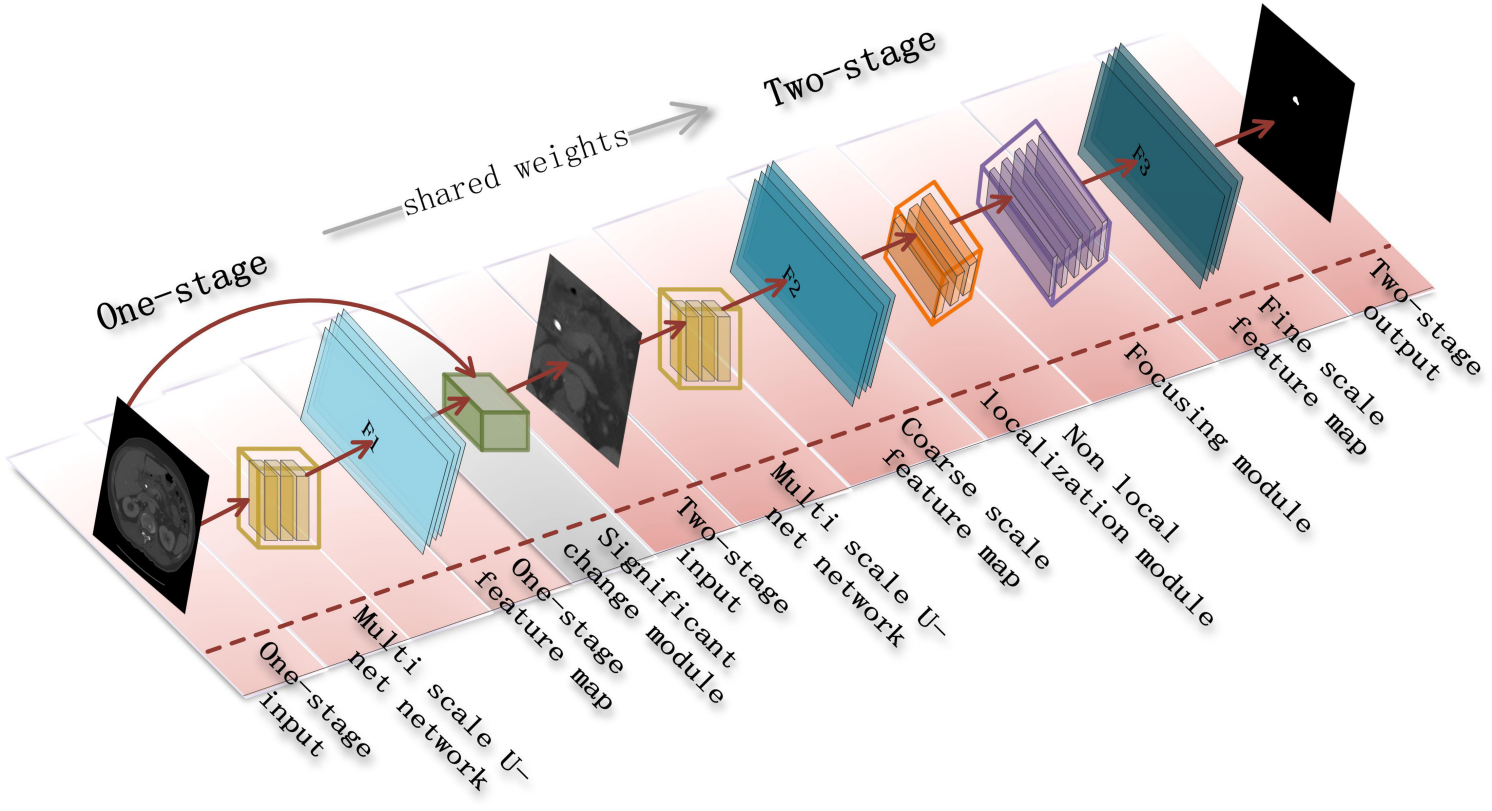
A two-stage cascade segmentation algorithm for pancreatic tumors.

Our goal is to accurately segment pancreatic tumors from a given abdominal CT scan. As shown in **Figure 2**, we design a cascade segmentation neural algorithm for segmenting pancreatic tumors. The algorithm contains two stages with a multi-scale U-Net as the backbone, where the first stage uses a multi-scale U-Net as the basic network to segment the approximate pancreatic region as the input for the second stage. In the second stage, we carefully design a multi-scale U-Net network based on non-local localization and focusing modules to segment pancreatic tumors to alleviate the problems of under-segmentation and false positives. In the training process, the small-scale pancreatic tumor is easily ignored due to its small contribution to the loss function, so we introduce an inter-class shared boundary-based metric for assisting the algorithm to obtain more robust parameters. We next detail the two stages and the loss function settings.

### 3.1 Multi-scale pancreas segmentation network

Pancreatic tumors, with variable scale and shape, and only a small part of the input data, bring certain difficulties to accurate segmentation. Pancreatic tumors are often closely related to the pancreas, so accurately locating the pancreas helps measure pancreatic tumors. Thus, we design a multi-scale pancreas segmentation network, which extracts and fuses multi-level features of the pancreas with the different sizes of convolutional kernels and connects with the network in the second stage by salient change modules.

#### 3.1.1 Multi-scale U-Net network

**Figure 3:**
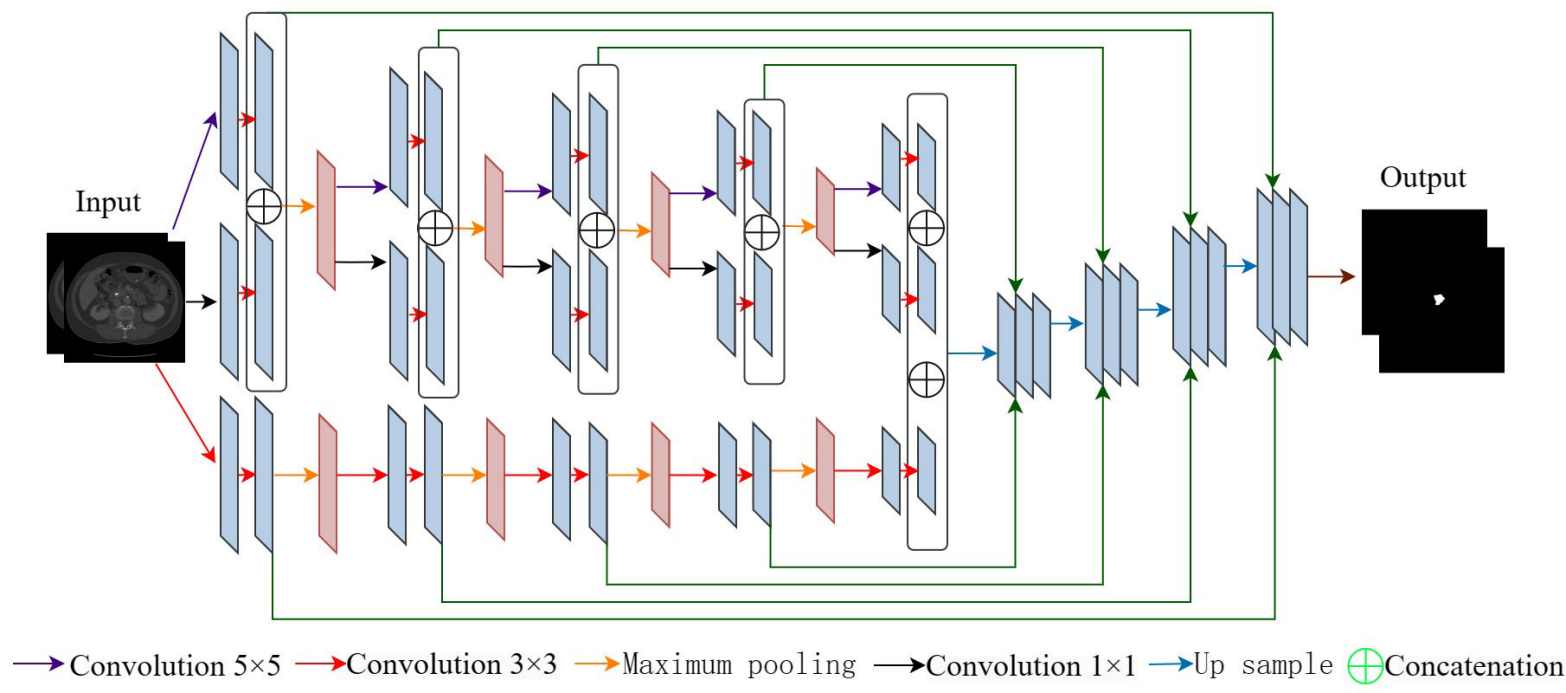
Architecture of multi-scale U-Net network.


We design a multi-scale U-Net network to extract and fuse contextual information from images at different scales, aiming to achieve high-precision pancreatic segmentation. As shown in **Figure 3**, each convolutional layer uses three convolutional kernels with different scales to extract features, which carry contextual information of multiple scales and retain fine-grained pancreatic position information. Multi-scale U-Net networks have a similar architecture to U-Net networks, both of which include a contraction and expansion stage with skip connections. Unlike U-Net with only one branch during the contraction phase, a multi-scale U-Net network consists of two encoder branches. In addition, the multi-scale U-Net network has two skip connections between the encoder and decoder, which allows the network to retain high-resolution features and transmit them to the decoder. Specifically, for the *i*-th block, we represent the outputs of its two encoders as *C_i_*_,1_ and *C_i_*_,2_, and the output of the next block *C_i_*_+1,1_ and *C_i_*_+1,2_ can be represented as Equations 1 and 2.

(1) $$C_{i+1,1}=p\left(conv_{3\times 3}\left(conv_{3\times 3}\left(C_{i,1}\right)\right)\right)$$

(2) $$C_{i+1,2}=p\left(conv_{3\times 3}\left(conv_{1\times 1}\left(C_{i,2}\right)\right)⊕conv_{3\times 3}\left(conv_{5\times 5}\left(C_{i,2}\right)\right)\right)$$

where $conv_{n\times n}$ represents the convolution operation with a kernel size of n×n. $C_{0,1}$ and $C_{0,2}$ represent the input image of the network, $C_{0,1}=C_{0,2}$ . p(⋅) represents the maximum pooling operation. ⊕ represents a concatenation operation. For the central layer, $C_{5,1}$ and $C_{5,2}$ can be represented as Equation 3.


(3) $$\begin{array}{ll} C_{5}=C_{5,1}=C_{5,2} & =conv_{3\times 3}\left(conv_{1\times 1}\left(C_{4,1}\right)\right)\\ & ⊕conv_{3\times 3}\left(conv_{3\times 3}\left(C_{4,2}\right)\right)\\ & ⊕conv_{3\times 3}\left(conv_{5\times 5}\left(C_{4,2}\right)\right) \end{array}$$

In this network, from the first block to the fourth block, each block is applied with three convolutional kernels of different sizes, namely 1×1, 3×3, 5×5, which can capture image features at three different scales. In general, when we perform downsampling operations, the feature dimension will decrease and some semantic information will be lost. To address this problem, we add a 1×1 convolutional kernel to the second branch of the encoder to enhance the model’s representation ability. In the decoder section, the original U-Net architecture copies features after the second convolutional layer of the encoder and connects them to the corresponding layer of the decoder. In our multi-scale U-Net network, the output of the first branch in the encoder is concatenated with the first convolution result of the corresponding decoder. The output of the second branch in the encoder is concatenated with the second convolution result of the decoder, and the features of the blocks in the two encoders are fused to enrich contextual information. If $U_{i}$ is the output of the *i*-th upsampled block, the output of the previous block can be expressed mathematically as Equation 4.


(4) $$U_{i-1}=conv_{3\times 3}\left(\left(conv_{3\times 3}\left(DeConv\left(U_{i}\right)⊕C_{i-1,1}\right)\right)⊕C_{i-1,2}\right)$$

where DeConv(·) represents deconvolution operation.

#### 3.1.2 Salient change module

*Cascade segmentation strategies* are widely used in scenarios with small target proportions in various fields. The existing methods (8, 30) obtain the approximate region of the target in the first stage as input for the second stage, and then finely segment the target in the second stage. The two stages of these methods are trained separately, meaning that the gradients in the second stage cannot update the network in the first stage. However, during testing, these two stages collaborate with each other in an iteration, resulting in inconsistent training and testing processes. What’s more, it is difficult to combine multi-level visual features into segmentation. For instance, the segmentation mask from the first stage, which carries rich feature information, is typically discarded except for the bounding box. This may result in poor convergence of the second stage network. To alleviate this disadvantage, we design a salient change module that connects the first and second stages. The salient change module uses the segmentation probability map from the first stage as prior information for the second stage, which can connect the two stages. During training, the first and second stages can be jointly optimized to improve their respective segmentation performance.

### 3.2 A multi-scale pancreatic tumor segmentation network based on non-local localization and focusing

**Figure 4:**
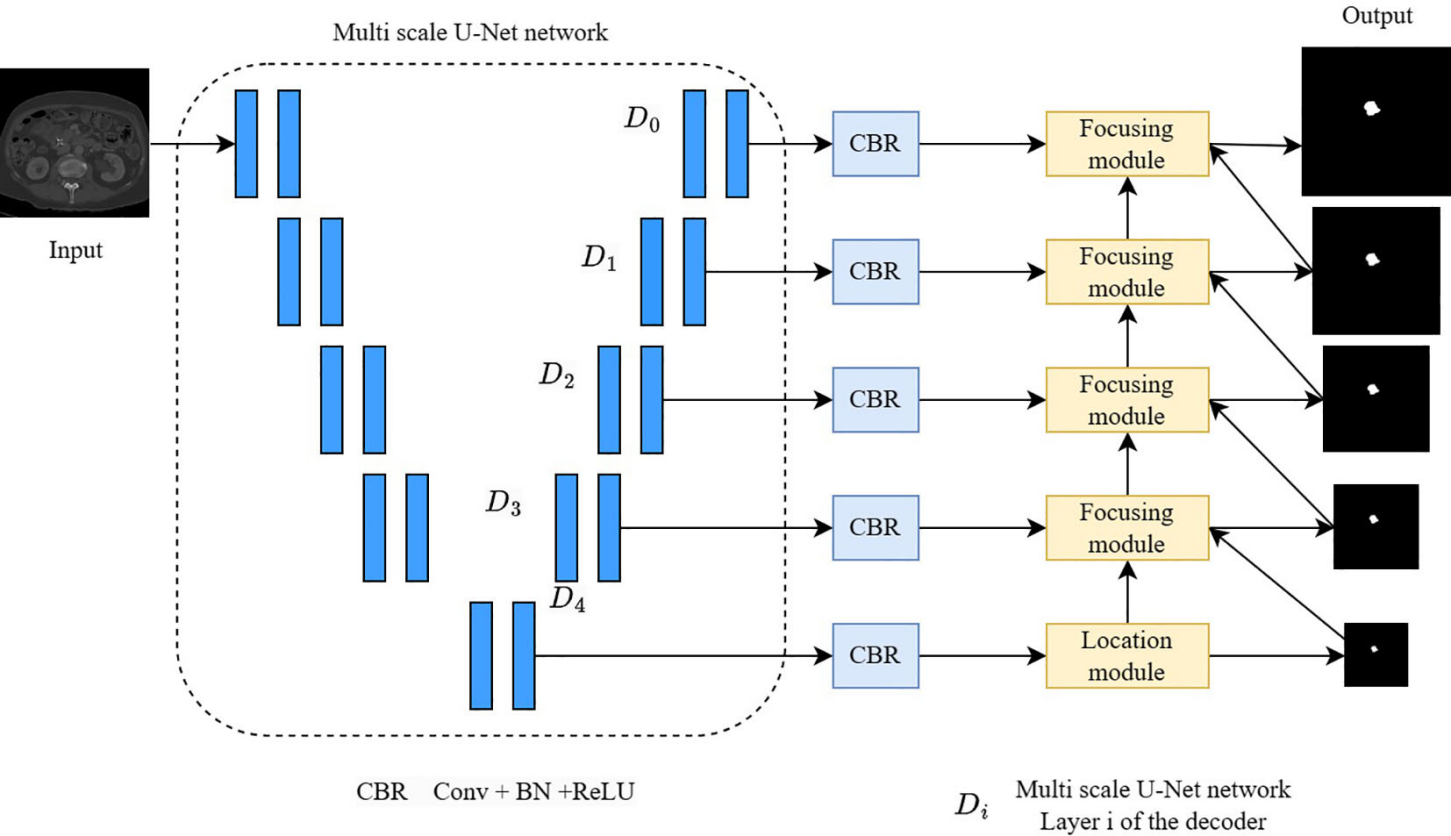
A multi-scale pancreatic tumor segmentation network based on non-local localization and focusing.

Pancreatic tumors have similar intensity and low contrast to the surrounding background in CT imaging and are highly correlated with camouflaged object detection and segmentation tasks. Inspired by the work of camouflaged object detection (31), we design a multi-scale pancreatic tumor segmentation network based on non-local localization and focusing modules on the second stage of the algorithm. Specifically, as shown in **Figure 4**, we first use a multi-scale U-Net encoder to extract multi-level features and input these features into five convolutional layers to reduce the number of channels. Then, a non-local localization module is added at the highest level of the encoder to locate the approximate area of the pancreatic tumor. Finally, a multiple focusing module is used to gradually detect and eliminate false negative and false positive interference. The input of the second stage is obtained by cropping the original image based on the segmentation probability map *P* output from the first stage.

A cropping function is defined, denoted as “crop”, and P is used as the reference image to binary it to Z. Pixels in Z that are not 0 are searched for, and the minimum 2D boundary including all pixels is calculated. The matrix is enlarged with a K-pixel wide margin, and crops the original coarse scale image accordingly. By reducing the input region in the second stage, the multi-scale pancreatic tumor segmentation network based on non-local localization and focusing can focus on smaller targets, preventing pancreatic tumors from being confused by the background region.

#### 3.2.1 Non-local localization module

The non-local localization module consists of a channel attention module and a spatial attention module, used to capture feature information between channels and spatial positions and to search for potential pancreatic tumors from a global perspective.

**Figure 5:**
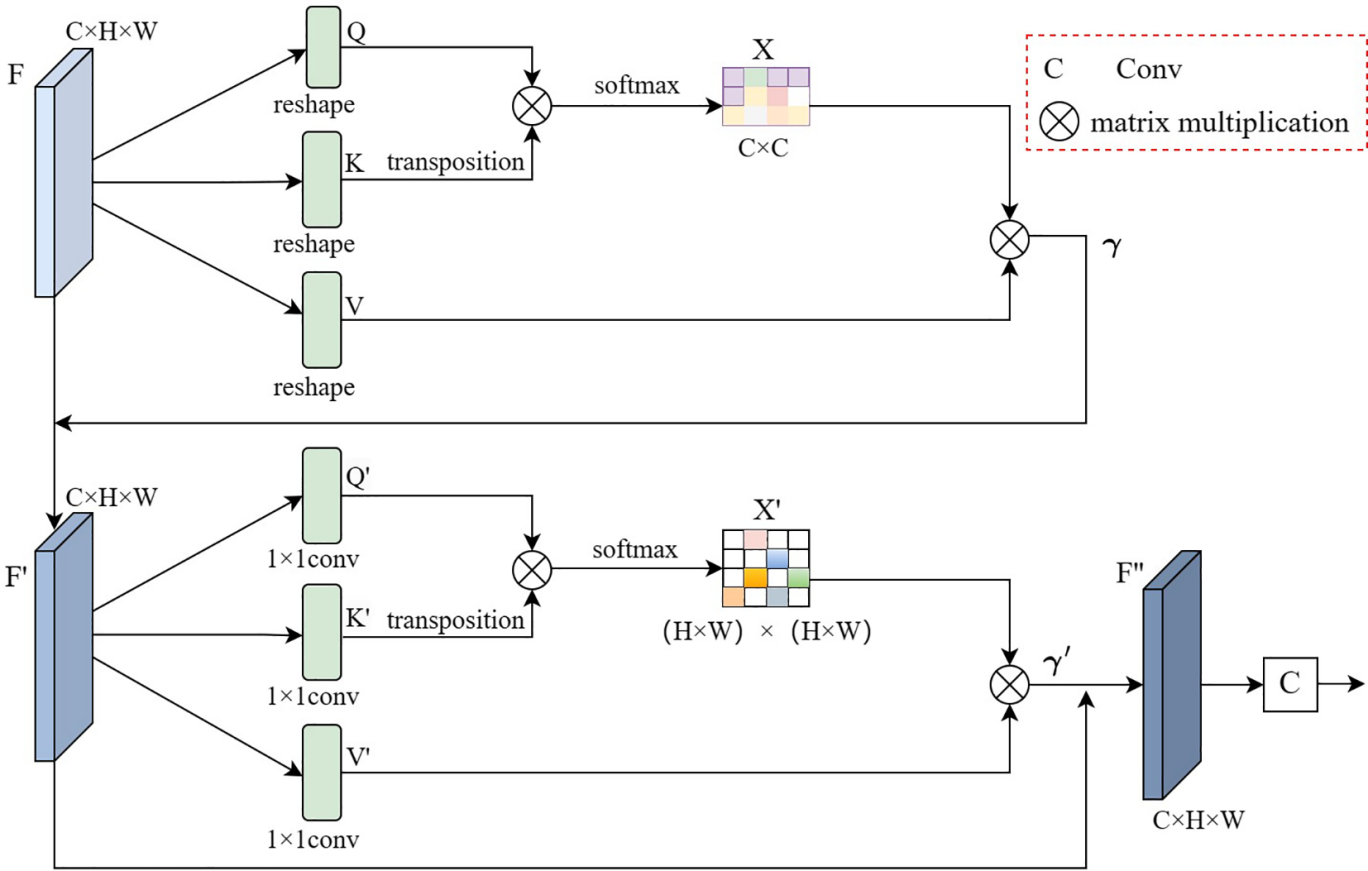
Illustration of non-local localization module.


The detailed structure of the non-local positioning module is shown in **Figure 5**. Given the top-level features of the multi-scale U-Net network decoder $F\in R^{C\times H\times W}$ , where *C* represents the number of channels, *H* represents height, and *W* represents the width, then the features *F* are reshaped to obtain queries *Q*, keys *K*, and values *V*, where $\left\{Q,K,V\right\}\in R^{C\times N},N=H\times W$ and *H*, *W* are all pixel values. Then matrix multiplication is performed on the transposition of *Q*, and *K*, and the channel attention map is calculated using the softmax function. The formula for the above process is shown in Equation 5.

(5) $$x_{i,j}=\frac{exp\left(Q_{i:}\cdot K_{j:}\right)}{\sum_{j=1}^{c}exp\left(Q_{i:}\cdot K_{j:}\right)}$$

where $Q_{i:}$ represents the *i*-th row of the matrix $Q,K_{j:}$ represents the *j*-th row of the matrix K , and $x_{i,j}$ represents the calculated impact of the *j*-th channel on the *i*-th channel. Then we perform Matrix multiplication between the matrix and the matrix, and the resulting matrix reshape is $R^{C\times H\times W}$ . Finally, in order to enhance fault tolerance, we multiply the obtained results by parameters γ and complete skip connection with the feature map F. The formula for the above process is shown in Equation 6.


(6) $$F_{i:}^{\prime }=\gamma \sum_{j=1}^{c}(x_{i,j}\cdot V_{j:})+F_{i:}$$

where γ is a learnable proportional parameter that gradually learns weights from the initial value, with an initial value of 1. The final feature $F^{\prime }$ can capture long-term semantic information between feature map channels, which is more recognizable than the initial feature F.

After obtaining the output feature $F^{\prime }$ of the channel attention mechanism, it will be used as the input for the spatial attention mechanism. First, we use three convolution kernels of 1×1 after inputting the features *F’*. The result of convolution is reshaped to obtain three new feature maps $Q^{\prime }$ , $K^{\prime }$ and $V^{\prime }$ , where $\left\{Q,K\right\}\in R^{C_{1}\times N},C_{1}=c/8,V^{\prime }\in R^{C\times N}$ . Then perform Matrix multiplication on the transposition of $Q^{\prime },K^{\prime }$ , and use the Softmax normalization function to calculate the spatial attention map $X^{\prime }\in R^{N\times N}$ . The formula for the above process is shown in Equation 7.


(7) $$x_{i,j}^{'}=\frac{exp\left(Q_{:i}^{'}\cdot K_{:j}^{'}\right)}{\sum_{j=1}^{N}exp\left(Q_{:i}^{'}\cdot K_{:j}^{'}\right)}$$

where $Q_{:i}^{\prime }$ represents the i-th column of the matrix *Q′*, $K_{:j}^{\prime }$ represents the j-th column of the matrix *K′*, and $x_{i,j}^{\prime }$ represents the calculated impact of the *j*-th position on the *i*-th position. Then we perform Matrix multiplication between the matrix $X^{\prime }$ and the matrix $V^{\prime }$ , and the resulting matrix is reshaped $R^{C\times H\times W}$ . Finally, similar to the channel attention mechanism, in order to enhance fault tolerance, we multiply the obtained results by the parameters $\gamma^{\prime }$ and complete skip connection with the feature map $F^{\prime }$ . The formula for the above process is shown in Equation 8.


(8) $$F_{i:}^{”}=\gamma^{\prime }\sum_{j=1}^{N}(V_{:j}^{\prime }\cdot x_{j,i}^{\prime })+F_{:i}^{\prime }$$

where $\gamma^{\prime }$ is gradually updated from the initial value, and the initial value is also set to 1. The final feature $F^{”}$ can capture the semantic correlation between feature map positions.

After obtaining the feature map $F^{”}$ , we connect a convolutional kernel with padding of 3 and size of 7×7 after $F^{”}$ to obtain the approximate area where the pancreatic tumor is located. Then the localization of the pancreatic tumor will be gradually improved by the focusing module.

#### 3.2.2 Focusing module

Due to the low contrast between some pancreatic tumors and the surrounding background, tumors segmented through the multi-scale U-Net network and the localization module often suffer from undersegmentation and false positives. To remove these erroneous segmentation errors, we perform contextual inference when predicting the target area (32). In order to detect false negative interference that is different from the determined background prediction area or false positive interference that is different from the determined foreground prediction area, we propose a focusing module. The focusing module takes the current layer features, higher-level features, and predictions of the multi-scale U-Net network decoder as inputs, and outputs more refined features and more accurate predictions.

**Figure 6:**
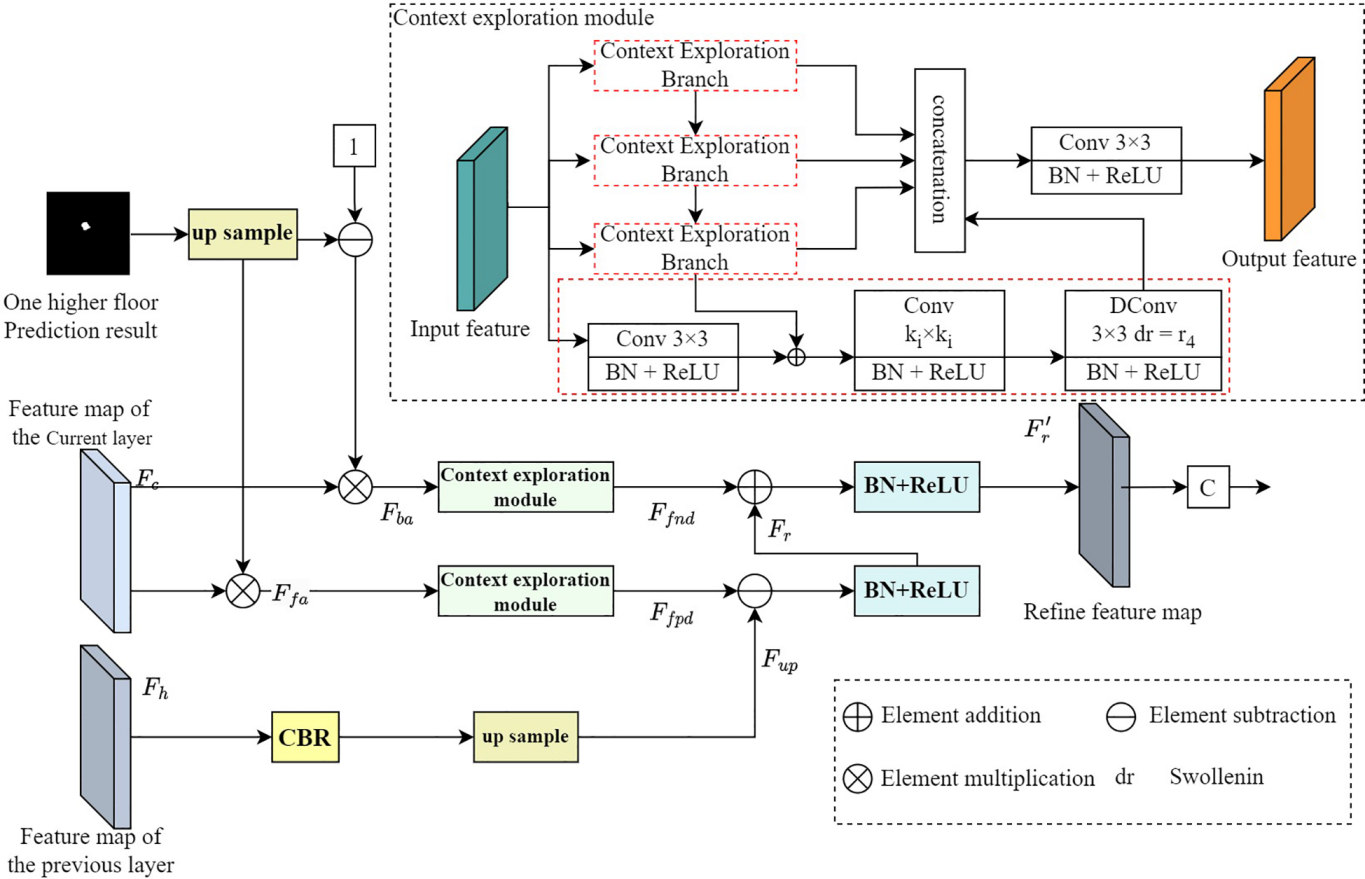
Structure diagram of focusing module.

The structure diagram of the focusing module is shown in **Figure 6**. Similar to the anti-attention mechanism (33), we first upsample the prediction results of the higher layer and normalize them using the softmax function. Then, we multiply the upsampling reverse and normalized results by the current layer’s features $F_{c}$ to obtain background $F_{ba}$ and foreground attention features $F_{fa}$ . Afterward, the features of these two parts are input into the context exploration module, and contextual reasoning is used to detect false negative interference *F_fnd_* and false positive interference $F_{fpd}$ .

The context exploration module mainly consists of four branches, each containing a convolution kernel of 3×3 which is used to reduce channels, a convolution kernel of *K_i_* ×*K_i_* which is used to extract local features, and a dilation convolution of 3×3 which is used to perceive contextual information. The dilation factor is *r_i_*, where i∈ {1,2,3,4}, *K_i_* is set to 1, 3, 5, 7, and $r_{i}$ is set to 1, 2, 4, 8. Each convolutional layer is connected to a BN layer and a ReLU nonlinear activation layer. Then we input the output of the i-th context exploration module into the i+1-th context exploration module, which can be further processed in a larger Receptive field, where i∈ {1,2,3}. Finally, these four branches are overlaid together on the channel dimension and fused through a 3×3 convolutional layer. With this design, contextual information can be perceived over a large range, enabling contextual reasoning and information mining. Following the context exploration module, false positives and false negatives are eliminated. The mathematical formulas of the context exploration module are shown in Equation 9.


(9) $$\begin{array}{ll} & F_{up}=U\left(CBR\left(F_{h}\right)\right)\\ & F_{r}=BR\left(F_{up}-\lambda F_{fpd}\right)\\ & F_{r}^{\prime }=BR\left(F_{r}+\gamma F_{fnd}\right) \end{array}$$

where $F_{h}$ represents the features of the higher input layer, CBR represents the combination of the convolutional layer, BN layer, and ReLU linear activation layer, U represents bilinear upsampling, $F_{r}^{\prime }$ represents finer features of the output, λ and γ are all learnable proportional parameters, with their initial values set to 1. Here, we use element-by-element subtraction to suppress ambiguous backgrounds (i.e. false positive interference) and element-by-element addition to supplement the identified foreground (i.e. false negative interference). Finally, convolutional layers are used on more refined features to obtain more accurate prediction maps. We use real annotations to supervise the generated prediction graph, forcing it to have a more accurate expression. This can guide the context exploration module to discover interference in the form of features, and then perform interference detection and removal.

### 3.3 Loss function and network training

During the entire training phase of the focusing module structure diagram, the update of network parameter weights depends on three parts: the segmentation loss of the pancreas in the first stage, the segmentation loss of the pancreatic tumor in the second stage, and the measurement loss based on shared boundaries between classes.

#### 3.3.1 Loss function of pancreas segmentation in the first stage

The first stage Loss function uses Dice loss, a widely used metric in medical image segmentation. The Dice loss function formula is shown in Equation 10.


(10) $$DiceLoss=1-\frac{2\left|X\cap Y\right|}{\left|X\right|+\left|Y\right|}$$

where *X* and *Y* represent the predicted results and the actual annotation respectively.

#### 3.3.2 Loss function of pancreatic tumor segmentation in the second stage

The second stage has five prediction outputs, one from the localization module and the other four from the focusing module. For the predicted output from the localization module, we use the combination of Binary Cross entropy loss (BCE Loss) and Intersection-over-union loss (IOU loss) as the loss function. Equation 11 to help the localization module locate the approximate area where the pancreatic tumor is located.


(11) $$L_{pm}=L_{bce}+L_{iou}$$

For the focusing module, our objective is to enhance attention towards areas prone to confusion, which are usually located at the boundary area and cavity of the tumor. Therefore, we use the combination of weighted BCE loss and weighted IOU loss as the loss function used in the focusing module. The loss function formula of the focusing module is shown in Equation 12.


(12) $$L_{fm}=L_{wbce}+L_{wiou}$$

The overall Loss function of the second stage can be expressed as Equation 13.


(13) $$L_{seg2}=L_{pm}+\sum_{i=2}^{5}2^{\left(4,i\right)}L_{fm}^{i}$$

where $L_{fm}^{i}$ is the loss predicted by the focusing module on the *i*-th layer of the multi-scale pancreatic tumor segmentation network based on non-local localization and aggregation.

Throughout the entire training process, the first stage needs to provide reasonable spatial weights and position information for the second stage, indicating that the segmentation in the first stage needs to be relatively accurate. However, in the early stages of training, the segmentation results are often inaccurate. Therefore, significant changes in the operational process of modules greatly affect the training effectiveness. We divide the training process into three steps: S, I, and J. This division is necessary because, during the early stages of training, the first stage may not accurately generate output, thereby limiting its ability to provide reliable segmentation results to the second stage. So, in the first step S, we turn off the saliency transformation module, optimize the two segmentation modules separately, and use real annotations as a reference to crop the image, which is used as input for the second stage. In the second step I, we start optimizing the saliency change module, allowing the spatial weights of the first stage to be transmitted to the second stage, while still using real annotations to control the cropping region. In step three J, we use the output of the second stage as a reference to control the cropping area, and the entire network is the same as in the testing stage.

#### 3.3.3 Loss function based on the measure of shared boundary between classes

The low proportion of small-scale pancreatic tumors in the Loss function seriously impacts the accurate segmentation of pancreatic tumors. In the commonly used pixel-by-pixel Cross entropy Loss function, the large target dominates the network training, while the small target contributes far less than the large target. Inspired by the measuring method based on the shared boundary between classes proposed by Guo et al. (34), we introduce this method to measure the spatial adjacency between each pair of object classes, and design a Loss function based on the measure of shared boundary between classes to solve the problem of small-scale targets.

We define the metric $m_{isb}$ based on shared boundaries between classes as a *n_c_* ×*n_c_* matrix, where $n_{c}$ is the number of divided categories. The metric based on shared boundaries between classes is calculated from the pancreatic tumor segmentation result graph *s*, where $s\left(x,y\right)\in \left\{1,\ldots ,n_{c}\right\}$ is the class label at the pixel points (*x,y*) in the segmentation graph. The value of *m_isb_* (*i,j*) is equal to the ratio of the length of the boundary shared between the *i*-th and *j*-th objects to the circumference of the *i*-th object. The $l_{i}$ represents the perimeter of the *i*-th object and the $l_{ij}$ represents the length of the shared boundary between the *i*-th and *j*-th objects. The formula for the (*i,j*)-th element in *m_isb_* can be expressed as Equation 14.


(14) $$m_{isb}\left(i,j\right)=\frac{l_{ij}}{l_{i}}$$

where $i=j=1,\ldots ,n_{c}$ , $m_{isb}\left(i,j\right)$ is equal to 0. Because the circumference of different objects is usually different, $m_{isb}$ is usually an asymmetric matrix. For the input image of 1×h×w , calculating the metric based on shared boundaries between classes for the segmentation prediction graph, the prediction graph of the segmentation network is a matrix $n_{c}\times h\times w$ , which is the probability that each pixel belongs to a class $c\in \left\{1,\ldots ,n_{c}\right\}$ . But when calculating the matrix $m_{isb}$ , the first step is to convert the predicted probability values into class labels, so that each pixel has only one class label and the boundary of the class can be determined. The category of pixel (x,y) is determined by the category corresponding to the maximum prediction probability. The mathematical formula for this process is shown in Equation 15.


(15) $$c^{*}=argmax_{c\in 1,\ldots ,n_{c}}s\left(c,x,y\right)$$

where s(c,x,y) is the probability that pixel points (x,y) are predicted as category c , $c^{*}$ represents the label corresponding to the maximum probability value.

In the Loss function based on the measure of shared boundary between classes, we use the mean squared error loss function. The mean squared error loss function formula is shown in Equation 16.


(16) $$L_{isb}=\frac{1}{nc\cdot nc}\sum_{i=1}^{nc}\sum_{j=1}^{nc}(m_{isb}^{pred}\left(i,j\right)-{\left(m_{isb}^{gt}\left(i,j\right)\right)}^{2}$$

where $m_{isb}^{pred}\left(i,j\right)$ is the predicted matrix of $m_{isb}$ , and $m_{isb}^{gt}\left(i,j\right)$ is the actual matrix of *m_isb_*.

#### 3.3.4 Overall loss function

The total loss function is the combination of the loss function based on the measure of the shared boundary between classes and the two-stage segmentation loss function:


(17) $$L=L_{seg1}+\alpha L_{seg2}+\beta L_{isb}$$

where $L_{seg1}$ and $L_{seg2}$ represent the Loss function of the first and second stages respectively, as well as *α* and *β* represent the proportion of each loss function respectively.

## 4 Experiment

### 4.1 Datasets and evaluation criteria

#### 4.1.1 Datasets

Following John Mongan et al. (35), we demonstrate the effectiveness and superiority of our algorithm on the pancreatic tumor dataset MSD and the pancreas dataset NIH. The code of our algorithm will be made public at https://github.com/HeyJGJu/Pancreatic-Tumor-SEG. Pancreatic tumor dataset MSD: this dataset comes from the pancreatic tumor segmentation dataset of the Medical Segmentation Decathlon (MSD) Challenge (36). It contains 281 available cases in NIFTI format with a resolution of 512 × 512, and the slice spacing is 5mm. Then we convert the NIFTI format to PNG, resulting in 2537 labeled sections, since there are 4 to 25 pancreatic tumor sections labeled by radiologists in each patient’s CT sequence. We apply 1957 images of 216 cases as the training sets and the remaining 580 images from 65 cases as the test sets. Pancreas dataset NIH: this dataset is developed by the US National Institute of Health (NIH) (37), which contains 82 available CT scans with a spatial resolution of 512 × 512 and the slice number varies from 181 to 466. We apply 5304 images of 62 cases as the training sets and the remaining 1699 images from 20 cases as the test sets.

#### 4.1.2 Evaluation criteria

Sørensen–Dice coefficient (Dice), sensitivity (SEN) and specificity (SPE) are used in our work. Among them, the mathematical formula of Dice indicator is shown in Equation 18.


(18) $$Dice=\frac{2\left|X\cap Y\right|}{\left|X\right|+\left|Y\right|}=\frac{2TP}{2TP+FP+FN}$$

where, *TP* represents true positives, indicating that the sample is identified as a pancreatic tumor and is indeed a pancreatic tumor; *FP* is false positives, signifying that the sample is identified as a pancreatic tumor when it is not actually a pancreatic tumor but rather background or other tissue; *FN* is false negatives, which means that the sample is determined not to be a pancreatic tumor, but actually it’s a pancreatic tumor. SEN represents the proportion of paired positive samples. The mathematical formula of SEN indicator is shown in Equation 19.


(19) $$SEN=\frac{A\cap B}{B}=\frac{TP}{TP+FN}$$

SPE and SEN are similar in that they represent the recognition ability of negative samples. The mathematical formula of SPE indicator is shown in Equation 20.


(20) $$SPE=\frac{A^{c}\cap B^{c}}{B^{c}}=\frac{TN}{FP+TN}$$

where *TN* is true negatives, which means that the sample is judged as the background and actually it is also the background pixel.

### 4.2 Implementation details

Our algorithm is implemented by PyTorch and trained on NVIDIA GeForce RTX 1080 GPU. We use multi-scale U-Net as the backbone for the pancreatic tumor segmentation branch and pancreatic tumor segmentation branch. In subsection 4.3.2, the training process is divided into three stages: S, I, and J. Stage S trains 2 epochs, Stage I trains 4 epochs, and Stage J trains 50 epochs. The batch size sets the size to 1. The optimizer selects SGD, the learning momentum is 0.9, the initial learning rate is set to 0.00001, and the weight attenuation is le-7. The hyperparameters *α* and *β* in Equation 17 are set to 0.9 and 0.4.

### 4.3 Comparisons with State-of-the-arts on MSD

#### 4.3.1 Quantitative evaluation

To verify the effectiveness and superiority of our proposed algorithm, 14 sets of experiments are conducted in this paper, which are compared with classical segmentation network models U-Net++ (38), Attention Unet (39), ResNet50 (40), Res_ UNet (41), U-Net (14), Dense-UNet (42), nnUnet (43), C2FNAS (44), V-NAS (45), and other existing tumor segmentation models HyperSegNAS (46), U-Shiftformer (47), DHT-Net (48) and IAG-Net (49). **Table 1** shows the detailed comparison results. The segmentation results of our proposed algorithm have the highest Dice value. Compared with the classic one-stage U-Net, the Dice value has increased by 10.11%, and compared with the currently best tumor segmentation model, the Dice value has increased by 1.62%. Overall, our algorithm has an increase in Dice values ranging from 1.62% to 16.35%, and the SPE value of the algorithm is also the highest, indicating that the algorithm can effectively solve the problem of false positives in pancreatic tumor segmentation. The SEN value has also improved compared to some models, but the overall SEN value is still low, indicating that there is still a problem of under-segmentation in the segmentation results. This may be due to unclear boundaries of pancreatic tumors and low contrast with the surrounding background.

**Table 1:** Comparison with state-of-the-art methods for pancreatic tumor segmentation on MSD dataset.

Method	Dice	SEN	SPE
U-Net++ (38)	0.4289	0.3966	0.9971
Attention Unet (39)	0.4317	0.5714	0.9977
ResNet50 (40)	0.4695	0.6056	0.9972
Res_UNet (41)	0.5264	0.7110	0.9960
U-Net (14)	0.4913	0.6846	0.9971
Dense-UNet (42)	0.5276	0.6133	0.9977
nnUnet (43)	0.5456	0.7268	0.9981
C2FNAS (44)	0.5636	0.6856	0.9982
V-NAS (45)	0.5730	0.6325	0.9942
U-Shiftformer (47)	0.5307	0.6833	0.9970
DHT-Net (48)	0.5618	0.6779	0.9975
HyperSegNAS (46)	0.5488	0.7123	0.9968
IAG-Net (49)	0.5762	**0.7342**	0.9969
Ours	0.5924	0.6824	0.9988

The bold value indicates the maximum value under the same indicator.

#### 4.3.2 Qualitative assessment

**Figure 7:**
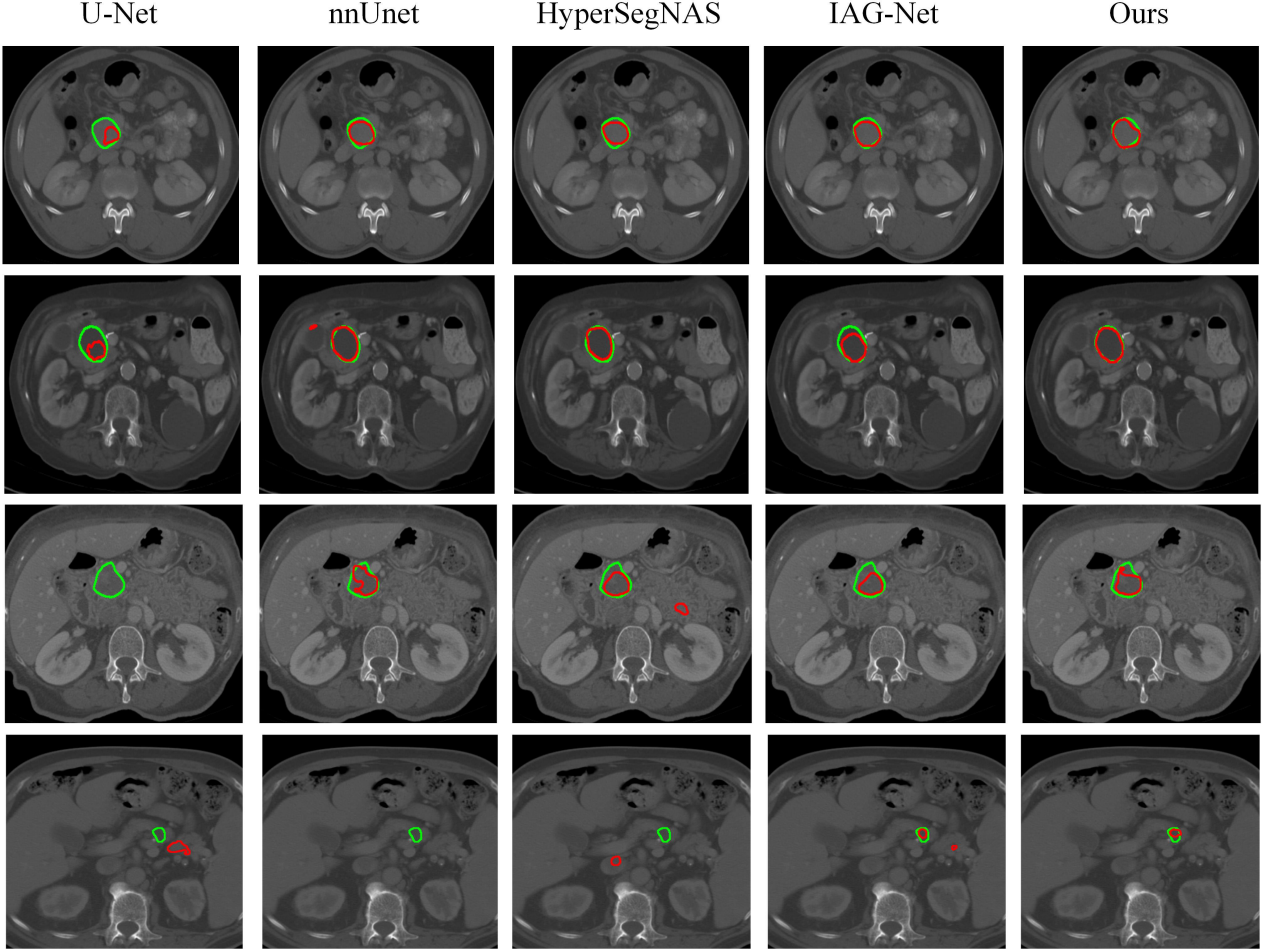
Visual comparison of pancreatic tumor segmentation produced by our algorithm and baseline networks.

The visual comparison of different methods on partial test image slices is shown in **Figure 7**. The green contour lines represent the predicted results of each network, and the red contour lines represent the true annotations. From the graph, it can be seen that U-Net and nnUnet often suffer from inaccurate localization. This is because small-scale pancreatic tumors have a small coverage range on the input image, resulting in less effective features learned by the network, and making the entire network insensitive to small-scale pancreatic tumors. Compared to U-Net and nnUnet, HyperSegNAS and IAG-Net networks have relatively better segmentation results compared to other networks, but some tumors still suffer from undersegmentation and false positives. This is because pancreatic tumors have a similar appearance to the background tissue and the boundaries are not clear. Therefore, when extracting features, the network is easily confused by chaotic similar regions in the background area, resulting in inaccurate segmentation. For these challenging issues, our algorithm is able to achieve segmentation edges that are very close to the true annotation of pancreatic tumors, resulting in more accurate segmentation of pancreatic tumors. This is because we design the segmentation network into two stages. By using the pancreatic prediction results in the first stage to shrink the input area of pancreatic tumors in the second stage and provide weight information for the second stage, we can more accurately locate the region where the pancreatic tumor is located. Simultaneously, multi-scale U-Net networks are used to extract contextual information and retain fine-grained information, making the localization of pancreatic and small-scale pancreatic tumors more accurate. In addition, we design a non-local localization module that can provide the focusing module with an approximate location of pancreatic tumors. In contrast, the focusing module can infer interference information from a complex background similar to surrounding tissues and eliminate these interferences. Thereby, the module can generate the real area where pancreatic tumors are located. Finally, the Loss function is improved by introducing a method based on the measure of the shared boundary between classes to increase the contribution of small-scale pancreatic tumors to the segmentation Loss function, improve the segmentation accuracy of pancreatic tumors, and make the segmentation boundary more consistent with the real labeled boundary. The experimental results once again demonstrate the superiority and accuracy of our proposed algorithm.

### 4.4 Comparisons with state-of-the-arts on NIH

We conduct 11 experiments to compare the effectiveness of our algorithm in pancreas segmentation with one-stage segmentation methods such as U-Net (14), Attention Unet (39), Nishio et al. (50), Li et al. (51), LMNS-Net (52) and the two-stage segmentation methods like Fixed-point (53), RSTN (54), Yu et al. (55), Zhang et al. (56), RTUNet (57). **Table 2** summarizes the results of the evaluation, obviously showing that the pancreatic segmentation task is challenging, and the Dice values of previous methods are relatively close. Overall, the two-stage segmentation method performs better than the one-stage segmentation method. Our algorithm in terms of Dice, achieves a score of 87.63% on the NIH. This is better than the best one-stage segmentation method LMNS-Net and the best two-stage segmentation method RTUNet, with improvements of 2.26% and 1.38% respectively. Additionally, our algorithm also outperforms other segmentation methods in terms of SEN and SPE.

**Table 2:** Comparison with state-of-the-art methods for pancreatic segmentation on NIH dataset.

Method	Dice	SEN	SPE
U-Net (14)	0.7470	0.5131	0.9964
Attention Unet (39)	0.8437	0.7764	0.9970
Nishio et al. (50)	0.7890	0.6883	0.9977
Li et al. (51)	0.8303	0.7207	0.9965
LMNS-Net (52)	0.8537	0.8115	0.9978
Fixed-point (53)	0.8257	0.6294	0.9968
RSTN (54)	0.8508	0.8466	0.9987
Yu et al. (55)	0.8453	0.8009	0.9972
Zhang et al. (56)	0.8490	0.7852	0.9975
RTUNet (57)	0.8625	0.8916	0.9981
**Ours**	**0.8763**	**0.9126**	**0.9988**

The bold value indicates the maximum value under the same indicator.

**Figure 8:**
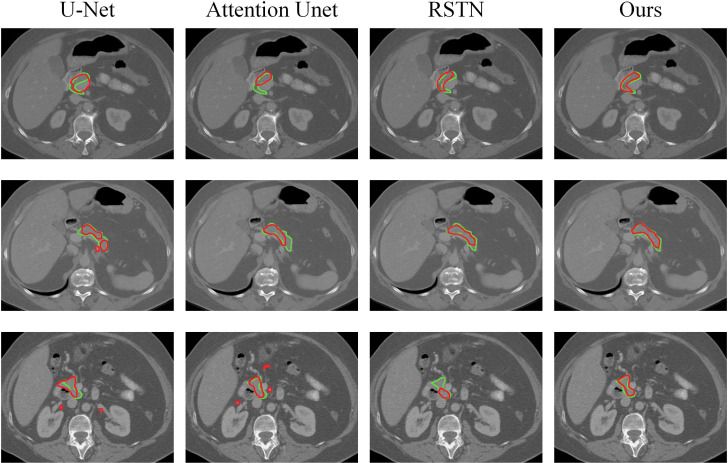
Visual comparison of pancreatic segmentation results produced by different methods.

In **Figure 8**, we show the visual segmentation results to intuitively compare with other state-of-the-art methods. It can be obviously shown that U-Net and Attention UNet are not accurate enough for pancreas localization due to their inability to learn effective features. RSTN is more accurate in capturing pancreas but edge segmentation is imprecise and segmentation results have false negatives. In contrast, our algorithm produces highly precise segmentation results that further verify the effectiveness of multi-scale U-Net in capturing finer details as well as the superiority of the non-local localization module and the focusing module in improving the performance of the algorithm.

### 4.5 Ablation studies

To verify the effectiveness of the different modules added, we conduct comprehensive ablation experiments on the MSD, using the Dice coefficient as the evaluation metric. All the experiments are done with the same settings for consistency.

#### 4.5.1 Effects of each module

In our segmentation model, the deep supervised information based on the pancreas in the first stage is added through the significance change module in the second stage. The traditional U-Net network is improved in the multi-scale pancreatic tumor segmentation network based on non-local localization and focusing, introducing multi-scale feature information, and adding a non-local localization module and focusing module. The design method and results of the ablation experiment are shown in **Table 3**. U represents the traditional U-Net network. M represents changing U-Net to a multi-scale U-Net network. P represents using the predicted results of pancreatic segmentation to reduce the input for pancreatic tumor network segmentation. T represents migrating the pancreatic segmentation model to the pancreatic tumor segmentation task during training. L and F represent adding the non-local localization module and the focusing module to the model respectively. Dice represents the Dice value of each segmentation model.

**Table 3:** Ablation study results.

Method	M	P	T	L	F	Dice
U						0.4913
M	✓					0.5234
M-P	✓	✓				0.5319
M-P-T	✓	✓	✓			0.5533
M-P-T-L	✓	✓	✓	✓		0.5651
M-P-T-L-F	✓	✓	✓	✓	✓	0.5924

We compare the quantitative results of our full pipeline and baseline networks on different settings. U, U-Net network; M, multi-scale U-Net network; P, using the predicted results of pancreatic segmentation to reduce the input for pancreatic tumor network segmentation; T, migrating the pancreatic segmentation model to the pancreatic tumor segmentation task during training; L, non-local localization module; F, focusing module.

Comparing the experimental results of U and M, the Dice value increases from 0.4913 to 0.5234. The performance comparison indicates the superiority of a multi-scale U-Net network over the traditional U-Net. The multi-scale U-Net effectively extracts and fuses information from different scales of images, enabling the network to segment tumors across various scales. The Dice value of the M-P experiment is 0.5319. This indicates that reducing the input scale of the pancreatic tumor segmentation model through the segmentation results of the pancreas can to some extent reduce the input of redundant information and prevent the network from being confused by background information. Compared with M-P, the Dice value of M-P-T in the experiment increased from 0.5319 to 0.5533, which proves the correlation between the pancreatic segmentation task and the pancreatic tumor segmentation task. The majority of pancreatic tumors appear in the pancreas. If the network masters the relevant features of the pancreas, it can help the network locate the location of pancreatic tumors more accurately and accelerate the training process of the network. After adding a non-local localization module to the model, the Dice value in the M-P-T-L experiment is 0.5651, which increases by 0.0118 compared to the M-P-T experiment. This proves that the non-local localization module can capture more detailed global feature information, and help the model locate the approximate range of tumors from a global perspective. Among all methods, the M-P-T-L-F experiment that fuses all modules yields the best results, with a Dice value of 0.5924. This is because the focus module detects and removes false positive and false negative interference through the context exploration module, thereby achieving more refined and accurate pancreatic tumor targets. The results of the M-P-T-L experiment and the M-P-T-L-F experiment reflect the effectiveness and design rationality of the non-local localization module and the focusing module.

**Figure 9:**
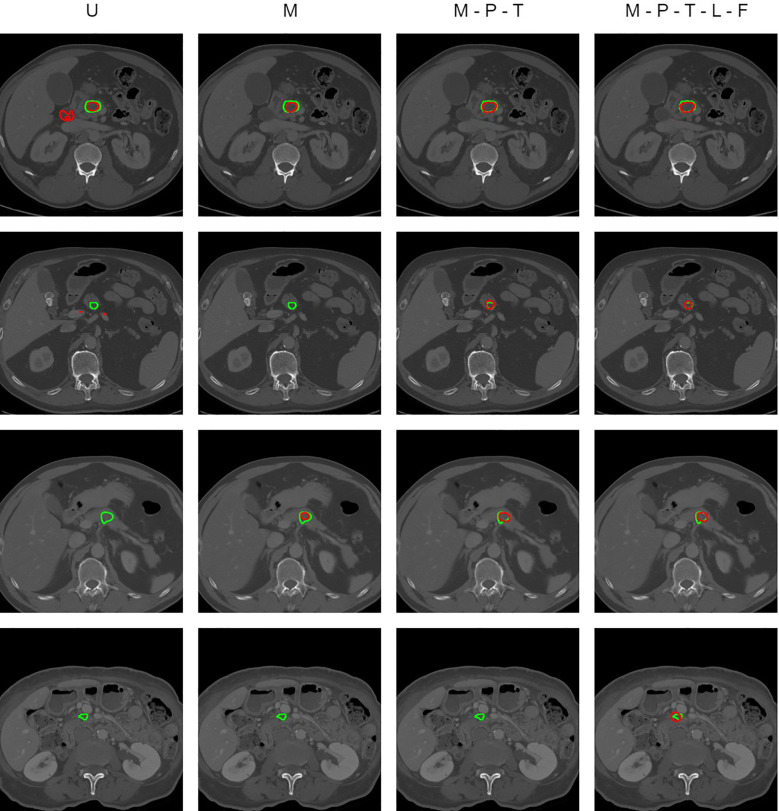
Visual comparison of pancreatic tumor segmentation produced by ablation study results.

**Figure 9** shows the prediction results of some CT image slices. It can be clearly seen that the segmentation results of pancreatic tumors based on the U-Net network and multi-scale U-Net network cannot accurately locate some small-scale and unclear boundaries of pancreatic tumors, and there are even omissions. In addition, those segmentation results often have the problem of undersegmentation or false positives. After introducing the deep supervised information based on the pancreas, more accurate position and weight information is provided for the segmentation of pancreatic tumors, which improves the problem of inaccurate localization of small-scale pancreatic tumors. M-P-T-L-F effectively solves the problems of undersegmentation and false positives by adding the non-local localization module and the focusing module. However, due to the low clarity and resolution of CT images on soft tissues, the images refined by the focusing module still have some degree of undersegmentation issues, especially inaccurate edge segmentation, such as in the fourth image slice.

#### 4.5.2 Effects of focusing module

**Figure 10:**
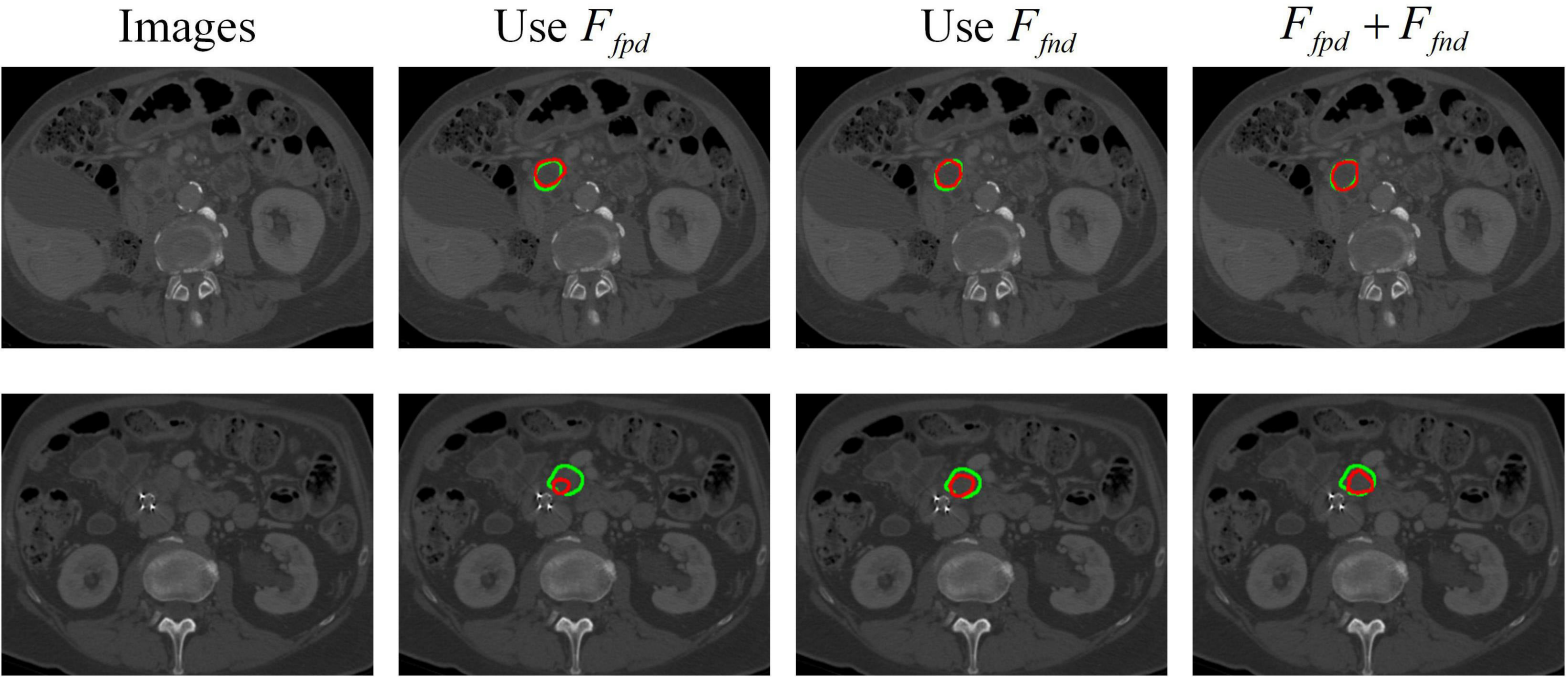
Visualization of segmentation results for pancreatic tumors using different interference information.

The findings presented in **Figure 10** demonstrate the effects of false-positive interference $\left(F_{fpd}\right)$ , false-negative interference $\left(F_{fnd}\right)$ , and both on the performance of our algorithm. The results clearly show that the combination of both types of interference yields the best segmentation results compared to only using *F_fpd_* or only using *F_fnd_*. This is because exploring interference information in features with discrimination helps the algorithm focus on easily confused areas, and pay close attention to tumor edges for contextual exploration, thereby achieving the finest edge segmentation.

## 5 Conclusions

In order to assist doctors in diagnosing pancreatic tumors and facilitate subsequent radiation therapy, we have designed a two-stage automatic segmentation algorithm for pancreatic tumors. The first stage uses a multi-scale network to segment the pancreas, and its segmentation results are used to reduce the input area of the second stage. The segmentation probability map of the first stage is used to provide prior information for the second stage. In the second stage, we develop a multi-scale network based on non-local localization and focusing on pancreatic tumor segmentation. We introduce a loss function based on shared boundary measures between classes to alleviate the problem of the low contribution of small-scale targets. Extensive experiments verify the effectiveness of our algorithm in pancreatic tumor segmentation tasks, outperforming other existing advanced segmentation methods. Our algorithm not only makes a certain contribution to the medical image community but also has a certain reference value for small target segmentation tasks in other fields.

## Data availability statement

The original contributions presented in the study are included in the article/**Supplementary Material**. Further inquiries can be directed to the corresponding author.

## Ethics statement

Ethical approval was not required for the studies involving humans in accordance with local legislation and institutional requirements because only publicly available pancreas and pancreatic tumor datasets were used.

## Author contributions

DQ: Writing – original draft, Writing – review & editing, Conceptualization, Data curation, Investigation, Methodology, Supervision, Validation, Visualization, Software. JJ: Writing – original draft, Writing – review & editing, Conceptualization, Data curation, Investigation, Methodology, Supervision, Validation, Visualization. SR: Conceptualization, Investigation, Visualization, Writing – review & editing. TZ: Investigation, Visualization, Writing – review & editing. HT: Funding acquisition, Supervision, Writing – review & editing. XT: Methodology, Supervision, Validation, Writing – review & editing. FX: Writing – review & editing.
