# Supplementary material for: A deep learning-based cascade algorithm for pancreatic tumor segmentation
Source: Front Oncol. 2024 Aug 7;14:1328146. doi: 10.3389/fonc.2024.1328146 (PMC11335681; doi:10.3389/fonc.2024.1328146)
Supplement: Supplementary file 1 [file DataSheet_1.pdf]

Detailed description about methods of Table 1

(1) U-Net++: UNet++ is a new, more powerful architecture for medical image segmentation. UNet++ is essentially a deeply-supervised encoder-decoder network where the encoder and decoder sub-networks are connected through a series of nested, dense skip pathways. The re-designed skip pathways aim at reducing the semantic gap between the feature maps of the encoder and decoder sub-networks. The article have evaluated UNet++ in comparison with U-Net and wide U-Net architectures across multiple medical image segmentation tasks: nodule segmentation in the low-dose CT scans of chest, nuclei segmentation in the microscopy images, liver segmentation in abdominal CT scans, and polyp segmentation in colonoscopy videos.

(2) Attention Unet: Attention Unet propose a novel attention gate (AG) model for medical imaging that automatically learns to focus on target structures of varying shapes and sizes. Models trained with AGs implicitly learn to suppress irrelevant regions in an input image while highlighting salient features useful for a specific task. This enables model to eliminate the necessity of using explicit external tissue/organ localisation modules of cascaded convolutional neural networks (CNNs). AGs can be easily integrated into standard CNN architectures such as the U-Net model with minimal computational overhead while increasing the model sensitivity and prediction accuracy. The proposed Attention U-Net architecture is evaluated on two large CT abdominal datasets for multi-class image segmentation. Experimental results show that AGs consistently improve the prediction performance of U-Net across different datasets and training sizes while preserving computational efficiency.

(3) ResNet50: Deeper neural networks are more difficult to train. This article present a residual learning framework to ease the training of networks that are substantially deeper than those used previously. This article explicitly reformulate the layers as learning residual functions with reference to the layer inputs, instead of learning unreferenced functions. ResNet50 provide comprehensive empirical evidence showing that these residual networks are easier to optimize, and can gain accuracy from considerably increased depth. On the ImageNet dataset, the article evaluate residual nets with a depth of up to 152 layers---8x deeper than VGG nets but still having lower complexity. An ensemble of these residual nets achieves 3.57% error on the ImageNet test set.

(4) Res UNet: Res UNet propose a novel volumetric convolutional neural network (ConvNet) with mixed residual connections. Extensive experiments on the open MICCAI PROMISE12 challenge dataset corroborated the effectiveness of the proposed volumetric ConvNet with mixed residual connections. Res UNet ranked the first in the challenge, outperforming other competitors by a large margin with respect to most of evaluation metrics. The proposed volumetric ConvNet is general enough and can be easily extended to other medical image analysis tasks, especially ones with limited training data.

(5) U-Net: There is large consent that successful training of deep networks requires many thousand annotated training samples. In this paper, UNet present a network and training strategy that relies on the strong use of data augmentation to use the available

annotated samples more efficiently. The architecture consists of a contracting path to capture context and a symmetric expanding path that enables precise localization. UNet show that such a network can be trained end-to-end from very few images and outperforms the prior best method (a sliding-window convolutional network) on the ISBI challenge for segmentation of neuronal structures in electron microscopic stacks.

(6) Dense-UNet: Recent work has shown that convolutional networks can be substantially deeper, more accurate, and efficient to train if they contain shorter connections between layers close to the input and those close to the output. Dense-UNet embrace this observation and introduce the Dense Convolutional Network (DenseNet), which connects each layer to every other layer in a feed-forward fashion. Whereas traditional convolutional networks with L layers have L connections--one between each layer and its subsequent layer--Dense-UNet has L(L+1)/2 direct connections. For each layer, the feature-maps of all preceding layers are used as inputs, and its own feature-maps are used as inputs into all subsequent layers. DenseNets have several compelling advantages: they alleviate the vanishing-gradient problem, strengthen feature propagation, encourage feature reuse, and substantially reduce the number of parameters. Dense-UNet evaluate network architecture on four highly competitive object recognition benchmark tasks (CIFAR-10, CIFAR-100, SVHN, and ImageNet). DenseNets obtain significant improvements over the state-of-the-art on most of them, whilst requiring less memory and computation to achieve high performance.

(7) nnUnet: nnU-Net is a deep learning framework that condenses the current domain knowledge and autonomously takes the key decisions required to transfer a basic architecture to different datasets and segmentation tasks. Without manual tuning, nnU-Net surpasses most specialised deep learning pipelines in 19 public international competitions and sets a new state of the art in the majority of the 49 tasks. The results demonstrate a vast hidden potential in the systematic adaptation of deep learning methods to different datasets.

(8) C2FNAS: C2FNAS propose a coarse-to-fine neural architecture search (C2FNAS) to automatically search segmentation network from scratch without inconsistency on network size or input size. Specifically, C2FNAS divide the search procedure into two stages: 1) the coarse stage, where C2FNAS search the macro-level topology of the network, i.e. how each convolution module is connected to other modules; 2) the fine stage, where C2FNAS search at micro-level for operations in each cell based on previous searched macro-level topology. The coarse-to-fine manner divides the search procedure into two consecutive stages and meanwhile resolves the inconsistency. C2FNAS evaluate our method on 10 public datasets from Medical Segmentation Decalthon (MSD) challenge, and achieve state-of-the-art performance with the network searched using one dataset, which demonstrates the effectiveness and generalization of our searched models.

(9) V-NAS: V-NAS propose to automatically search the network architecture tailoring to volumetric medical image segmentation problem. Concretely, V-NAS formulate the structure learning as differentiable neural architecture search, and let the network itself choose between 2D, 3D or Pseudo-3D (P3D) convolutions at each layer.

V-NAS evaluate method on 3 public datasets, i.e., the NIH Pancreas dataset, the Lung and Pancreas dataset from the Medical Segmentation Decathlon (MSD) Challenge. V-NAS consistently outperforms other state-of-the-arts on the segmentation tasks of both normal organ (NIH Pancreas) and abnormal organs (MSD Lung tumors and MSD Pancreas tumors), which shows the power of chosen architecture. Moreover, the searched architecture on one dataset can be well generalized to other datasets, which demonstrates the robustness and practical use of our proposed method.

(10) U-Shiftformer: U-Shiftformer is a network structure based on the shifted attention mechanism to overcome the limitation of existing convolution neural networks (CNNs) in brain tumor segmentation that lacks multimodal information interaction. The U-Shiftformer takes the U-shape encoder-decoder structure as the backbone and embeds the proposed Shiftformer module to exchange the information between modalities in the downsampling process. The Shiftformer module contains one standard attention, and three proposed shifted attention modules, in which the shifted attention module considers the information exchange by constructing the relationship between adjacent modalities. Experiments compare the proposed U-Shiftformer with SOTA networks in the dice, precision, and Hausdorff metrics. Its average accuracies of these metrics surpass all the comparison networks and achieve 0.8424, 0.8675, 0.9244, and 1.2961 in dice, precision, sensitivity, and Hausdorff metrics, respectively.

(11) DHT-Net: To learn and extract complex tumor features of varied tumor size, location, and morphology for more accurate segmentation, this article propose a Dynamic Hierarchical Transformer Network, named DHT-Net. The DHT-Net mainly contains a Dynamic Hierarchical Transformer (DHTrans) structure and an Edge Aggregation Block (EAB). The DHTrans first automatically senses the tumor location by Dynamic Adaptive Convolution, which employs hierarchical operations with the different receptive field sizes to learn the features of various tumors, thus enhancing the semantic representation ability of tumor features. Then, to adequately capture the irregular morphological features in the tumor region, DHTrans aggregates global and local texture information in a complementary manner. In addition, DHT-Net introduce the EAB to extract detailed edge features in the shallow fine-grained details of the network, which provides sharp boundaries of liver and tumor regions. Article evaluate DHT-Net on two challenging public datasets, LiTS and 3DIRCADb.

(12) HyperSegNAS: To enable one-shot NAS for medical image segmentation, HyperSegNAS introduces a HyperNet to assist super-net training by incorporating architecture topology information. Such a HyperNet can be removed once the super-net is trained and introduces no overhead during architecture search. Experiment show that HyperSegNAS yields better performing and more intuitive architectures compared to the previous state-of-the-art (SOTA) segmentation networks; furthermore, it can quickly and accurately find good architecture candidates under different computing constraints. HyperSegNAS is evaluated on public datasets from the Medical Segmentation Decathlon (MSD) challenge, and achieves SOTA performances.

(13) IAG-Net: This article propose an Inductive Attention Guidance Network

(IAG-Net) to jointly learn a global image-level classifier for normal/PDAC (Pancreatic ductal adenocarcinoma) classification and a local voxel-level classifier for semi-supervised PDAC segmentation. IAG-Net instantiate both the global and the local classifiers by multiple instance learning (MIL), where the attention guidance, indicating roughly where the PDAC regions are, is the key to bridging them: For global MIL based normal/PDAC classification, attention serves as a weight for each instance (voxel) during MIL pooling, which eliminates the distraction from the background; For local MIL based semi-supervised PDAC segmentation, the attention guidance is inductive, which not only provides bag-level pseudo-labels to training data without per-voxel annotations for MIL training, but also acts as a proxy of an instance-level classifier. Experimental results show that our IAG-Net boosts PDAC segmentation accuracy by more than 5% compared with the state-of-the-arts.

Detailed description about methods of Table 2

(1) U-Net: There is large consent that successful training of deep networks requires many thousand annotated training samples. In this paper, UNet present a network and training strategy that relies on the strong use of data augmentation to use the available annotated samples more efficiently. The architecture consists of a contracting path to capture context and a symmetric expanding path that enables precise localization. UNet show that such a network can be trained end-to-end from very few images and outperforms the prior best method (a sliding-window convolutional network) on the ISBI challenge for segmentation of neuronal structures in electron microscopic stacks.
(2) Attention Unet: Attention Unet propose a novel attention gate (AG) model for medical imaging that automatically learns to focus on target structures of varying shapes and sizes. Models trained with AGs implicitly learn to suppress irrelevant regions in an input image while highlighting salient features useful for a specific task. This enables model to eliminate the necessity of using explicit external tissue/organ localisation modules of cascaded convolutional neural networks (CNNs). AGs can be easily integrated into standard CNN architectures such as the U-Net model with minimal computational overhead while increasing the model sensitivity and prediction accuracy. The proposed Attention U-Net architecture is evaluated on two large CT abdominal datasets for multi-class image segmentation. Experimental results show that AGs consistently improve the prediction performance of U-Net across different datasets and training sizes while preserving computational efficiency.
(3) Nishio et al.: Combinations of data augmentation methods and deep learning architectures for automatic pancreas segmentation on CT images are proposed and evaluated. Images from a public CT dataset of pancreas segmentation were used to evaluate the models. Baseline U-net and deep U-net were chosen for the deep learning models of pancreas segmentation. Methods of data augmentation included conventional methods, mixup, and random image cropping and patching (RICAP). Ten combinations of the deep learning models and the data augmentation methods were evaluated. Four-fold cross validation was performed to train and evaluate these models with data augmentation methods. The dice similarity coefficient (DSC) was calculated between automatic segmentation results and manually annotated labels and

these were visually assessed by two radiologists. The performance of the deep U-net was better than that of the baseline U-net with mean DSC of 0.703 – 0.789 and 0.686 – 0.748, respectively. In both baseline U-net and deep U-net, the methods with data augmentation performed better than methods with no data augmentation, and mixup and RICAP were more useful than the conventional method. The best mean DSC was obtained using a combination of deep U-net, mixup, and RICAP, and the two radiologists scored the results from this model as good or perfect in 76 and 74 of the 82 cases.

(4) Li et al.: A novel multi-level pyramidal pooling residual U-Net with adversarial mechanism was proposed for organ segmentation from medical imaging, and was conducted on the challenging NIH Pancreas-CT dataset. In order to achieve accurate segmentation, model frstly involved residual learning into an adversarial U-Net to achieve a better gradient information fow for improving segmentation performance. Then, model introduced a multi-level pyramidal pooling module (MLPP), where a novel pyramidal pooling was involved to gather contextual information for segmentation, then four groups of structures consisted of a diferent number of pyramidal pooling blocks were proposed to search for the structure with the optimal performance, and two types of pooling blocks were applied in the experimental section to further assess the robustness of MLPP for pancreas segmentation. For evaluation, Dice similarity coefcient (DSC) and recall were used as the metrics in this work.

(5) LMNS-Net: In the study and research of medical images, the sharp and smooth pancreatic segmentation challenge is a critical and challenging one. The most widely utilized and effective technique for pancreatic segmentation with smooth and precise results is a proposed LMNS-net deep learning, bottom-up approach. The proposed LMNS-net is used to automatically segment the pancreas in clinical abdominal computed tomography (CT) images. For the segmentation of the acute pancreas using several angles of CT scans, such as coronal, axial, and sagittal, a proposed LMNS-net model is used. In the LMNS-Net model, 12 layers are used with 4 convolution layers. LMNS-Net model is used for many organ segmentation from CT scans clinical images with high accuracy. The lightweight multiscale block is used in the proposed approach which is aggregating the required feature only so unused information dropout at the convolution layer. The computation time-period is reduced as related to the state-of-art. Validation is 99.78% and loss values vary from 1 to 0 only. The LMNS-Net model achieved a dice similarity index score up to 88.68 ± 57.49%. The LMNS-Net model takes 1 to 3 s for the segmentation of medical CT images in the testing process. The LMNS-Net model takes very less time for testing purposes as compared to other approaches. Top-down approaches are not useful in the detection of pancreatic cancer images from CT scan images. In Bottom-Up approaches, the LMNS-Net is useful to detect accurate sharpness of the pancreas and kidney, the pancreatic cancer-affected area within less time only.

(6) Fixed-point: Deep neural networks have been widely adopted for automatic organ segmentation from abdominal CT scans. However, the segmentation accuracy of some small organs (e.g., the pancreas) is sometimes below satisfaction, arguably because

deep networks are easily disrupted by the complex and variable background regions which occupies a large fraction of the input volume. This article formulate this problem into a fixed-point model which uses a predicted segmentation mask to shrink the input region. This is motivated by the fact that a smaller input region often leads to more accurate segmentation. In the training process, Fixed-point use the ground-truth annotation to generate accurate input regions and optimize network weights. On the testing stage, Fixed-point fix the network parameters and update the segmentation results in an iterative manner. Experiment evaluate Fixed-point on the NIH pancreas segmentation dataset, and outperform the state-of-the-art by more than 4%, measured by the average Dice-Sørensen Coefficient (DSC).

(7) RSTN: This article allow a gradual optimization to improve the stability of the recurrent saliency transformation network (RSTN), and introduce a hierarchical version named H-RSTN to segment tiny and variable neoplasms such as pancreatic cysts. Experiments are performed on several CT datasets including a public pancreas segmentation dataset, own multi-organ dataset, and a cystic pancreas dataset. In all these cases, the RSTN outperforms the baseline (a stage-wise coarse-to-fine approach) significantly. Confirmed by the radiologists in our team, these promising segmentation results can help early diagnosis of pancreatic cancer.

(8) Xie et al.: This articial aim at segmenting small organs (e.g., the pancreas) from abdominal CT scans. As the target often occupies a relatively small region in the input image, deep neural networks can be easily confused by the complex and variable background. To alleviate this, researchers proposed a coarse-to-fine approach, which used prediction from the first (coarse) stage to indicate a smaller input region for the second (fine) stage. Despite its effectiveness, this algorithm dealt with two stages individually, which lacked optimizing a global energy function, and limited its ability to incorporate multi-stage visual cues. Missing contextual information led to unsatisfying convergence in iterations, and that the fine stage sometimes produced even lower segmentation accuracy than the coarse stage. This paper presents a Recurrent Saliency Transformation Network. Experiments in the NIH pancreas segmentation dataset demonstrate the state-of-the-art accuracy, which outperforms the previous best by an average of over 2%. Much higher accuracies are also reported on several small organs in a larger dataset collected by ourselves.

(9) Zhang et al.: This paper establishes a novel end-to-end DCNN model for pursuing high-accurate automatic pancreas segmentation but with low computational cost. Specifically, built upon a simplified FCN architecture, paper propose two novel network modules, named as the scale-transferrable feature fusion module (STFFM) and prior propagation module (PPM), respectively, for pancreas segmentation. Equipped with the scale-transferrable operation, STFFM can learn rich fusion features but with very lightweight network architecture. By dynamically adapting the spatial prior to the input slice data as well as the deep feature maps, PPM enables the network model to explore informative spatial priors for pancreas segmentation. Comprehensive experiments on the NIH dataset and the MSD dataset are conducted to evaluate the proposed approach. The obtained experimental results demonstrate that this approach can effectively reduce the computational cost and simultaneously

archive the outperforming performance when compared to the state-of-the-art methods.

(10) RTUNet: Accurate pancreas segmentation is crucial for the diagnostic assessment of pancreatic cancer. However, large position changes, high variability in shape and size, and the extremely blurred boundary make the task of pancreas segmentation challenging. To alleviate these challenges, this article propose the residual transformer UNet (RTUNet) to fit the nature of the pancreas. Specifically, a residual transformer block is implemented to extract multi-scale features from a global perspective which captures high variabilities in the pancreas position. In addition, a dual convolutional down-sampling strategy is leveraged to obtain precise shape and size features of the pancreas in a large receptive field which prevents the loss of information. RTUNet finally propose a dice hausdorff distance loss that makes the network focus on the pancreas boundary. Through extensive experiments on the public NIH dataset, RTUNet achieved a dice similarity coefficient (DSC) of 86.25%, which outperforms the state-of-the-art DSC of 85.49%.
